# Mxenes Derived Laminated and Magnetic Composites with Excellent Microwave Absorbing Performance

**DOI:** 10.1038/s41598-019-40336-9

**Published:** 2019-03-08

**Authors:** Wanlin Feng, Heng Luo, Yu Wang, Sifan Zeng, Yongqiang Tan, Lianwen Deng, Xiaosong Zhou, Haibin Zhang, Shuming Peng

**Affiliations:** 1 Innovation Research Team for Advanced Ceramics, Institute of Nuclear Physics and Chemistry, China Academy of Engineering Physics, Mianyang, 621900 China; 2 School of Physics and Electronics, Institute of Super-microstructure and Ultrafast Process in Advanced Materials, Central South University, Changsha, 410083 China

## Abstract

Two dimensional materials have been widely identified as promising microwave absorbers, owing to their large surface area and abundant interfaces. Here, a novel laminated and magnetic composite derived from Mxene was designed and successfully synthesized via facile hydrothermal oxidization of nickel ion intercalated Ti_3_C_2_. Highly disordered carbon sheets were obtained by low temperature hydrothermal oxidization, and the *in-situ* produced TiO_2_ and NiO nanoparticles embedded closely between them. This layered hybrid exhibits excellent microwave absorbing performance with an effective absorbing bandwidth as high as 11.1 GHz (6.9–18 GHz) and 9 GHz (9–18 GHz) when the thickness is 3 and 2 mm, respectively. Besides the high dielectric loss, magnetic loss and ohmic loss of the composite, the amorphous nature of obtained carbon sheets and multi-reflections between them are believed to play a decisive role in achieving such superior microwave absorbing performance.

## Introduction

With the ever faster development of electrical communication tools, a substantial increase of hazard electromagnetic pollution has become a severe problem in both civil and military applications. Therefore, broadband and highly efficient electromagnetic wave (EMW) absorbing materials are greatly demanded to minimize the adverse influences in aspects of healthcare, electrical devices and national defense security^1–3^. Great efforts have been devoted to the development of high performance EMW absorbers with a broadband microwave absorbing (MA) ability, especially in the radar frequency range of 2–18 GHz. Generally, materials with laminated structure are well known as promising microwave absorbers, and the unique layered structure with large surface area and heterogeneous interfaces which could produce abundant defects accompanying polarizations and interface scattering, is thought to be the main contribution to the outstanding MA ability^3–5^. Specially, graphene-based 2D MA materials are the most widely studied in the past decade. Although pure graphene or reduced graphene oxide (RGO) sheets exhibit poor MA ability^6^, their MA performance can be significantly improved by assembling some functional particles with G or RGO, such as G/Ni^7,8^, RGO/Fe_x_O_y_^9,10^, RGO/polymer^11^, and RGO/CNTs^12^. Nevertheless, the assembling will inevitably lead to extensive agglomeration, which could greatly limit the enhancement of the MA efficiency^10^. Besides, the carbon atoms of graphene are highly ordered and the carbon layers are easily to restack, leading to a significant decrease of MA ability as well^6^.

Mxenes are a new series of two dimensional materials with the formula M_n+1_X_n_T_x_, where M is an early transition metal, X is carbon and/or nitrogen, T_x_ is the surface terminations usually -OH and/or –F^13^. Owing to the laminated morphology and unique combination of electrical conductivity and hydrophilicity, Mxenes are well known to be attractive candidates for various applications^14–20^, as well as electromagnetic interference (EMI) shielding/absorbing materials^21–24^. In 2016, Y. C. Qing *et al*.^22^ exhibited the superior microwave absorbing capability in frequency range of 12.4–18 GHz. In our previous work^23^, we also demonstrated the excellent microwave absorbing performance of Ti_3_C_2_ Mxene enabled microwave absorber within frequency range of 11.2–18 GHz, and the underlying mechanisms of dielectric responses were intensively discussed. M. Han *et al*.^24,25^ reported the enhanced microwave absorbing performance of modified Ti_3_C_2_ Mxene and its derives in 2016 and 2017, respectively, which opened a new way to the study of high performance microwave absorbing materials.Ti_3_C_2_ as a representative of Mxene family who possess active surface functional terminations, which could give rise to remarkable defects polarizations was proved to be responsible for the promising MA ability. Besides, the divers surface modifications of Mxene by ion intercalation, annealing and oxidization could offer tunable microwave absorbing properties^24,25^.

The high electrical conductivity of Ti_3_C_2_ is one big obstacle for the application as MA materials which should have intensified polarizations and moderate conductivity. Hydrothermal treatment of Ti_3_C_2_ will derive oxidized MXene (MO), a kind of titania–carbon hybrid material^26^. It should be noted that the introduction of oxidation will greatly decrease the conductivity and increase the polarization defects within Ti_3_C_2_, which seems to be an effective approach to enhance the MA properties of Ti_3_C_2_. What is more, numerous reports have confirmed that carbon nanostructure with the incorporation of magnetic particles can effectively improve the EMW absorbing capacity of composites^27,28^. Therefore, it is highly expected that incorporating magnetic species into oxidized Mxene could be an effective route to obtain high performance EMW absorbing materials.

Herein, we demonstrate a facile synthesis route towards the preparation of amorphous carbon supported laminated and magnetic hybrids (denoted as NiO&TiO_2_@C) by the hydrothermal oxidation of nickel ions intercalated Ti_3_C_2_. The relatively low temperature (180 °C) of hydrothermal oxidation makes the as-formed atomically thin carbon sheets highly disordered, and the *in-situ* oxidized TiO_2_ and NiO nanoparticles embedded closely into each carbon layers to form a sandwich-like structure. The structure, magnetic, microwave electromagnetic responses and absorbing properties of the as-prepared composites were investigated. The results indicate that the composites exhibit excellent MA performance due to multi-mechanisms of microwave attenuation. Furthermore, our proposed method provides an important guideline to insert other metal ions into Mxenes and to derive other metal oxide hybrids, which expands the applications of Mxenes.

## Results and Discussion

### Composition and microstructure

**Figure 1:**
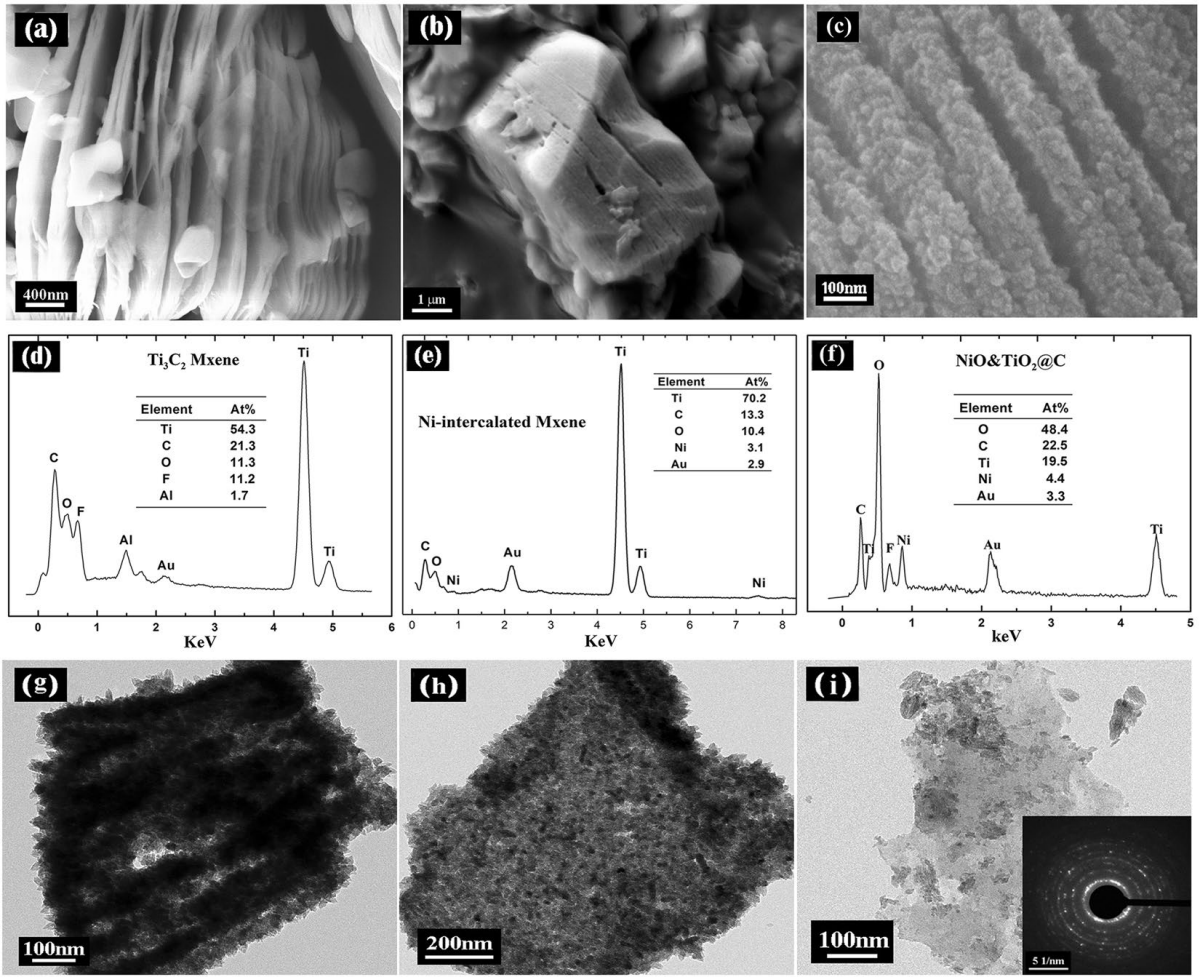
Microstructure of Mxene, Ni-intercalated MXene and Derived Composites: (**a–c**) SEM image of typical laminated structure of Ti_3_C_2_, Ni-intercalated MXene and the derived composites; (**d–f**) EDS spectrum of Ti_3_C_2_, Ni-intercalated MXene and the derived composites; (**g**) TEM image of cross section of a NiO&TiO_2_@C particle, showing the typical sandwich-like structure; (**h**) TEM image of a single-layer composite of NiO&TiO_2_@C, showing a monolayer carbon sheet coated with plenty of oxide particles; (**i**) a monolayer carbon sheet coated with few oxide particles, inset shows SAED pattern. (EDS spectrums are prepared with Origin (OriginLab, Northampton, MA)).

Figure 1(a) shows the typical morphology of Ti_3_C_2_ with an accordion-like structure. Aluminum atoms are extracted and the sonication makes the layered structure swelled. Energy dispersive X-ray spectroscopy (Fig. 1(d)) reveals that the laminated materials are composed of Ti, C, O and F. The excess O and F signal could be ascribed to the surface terminations (-F and/or -OH), and possibly the intercalated water and hydrofluoric acid between Ti_3_C_2_ layers^13^. After Ni^2+^ intercalation, MXene still keeps the lamellar morphology, but the inner space of MXene most likely is filled with Ni ions (as shown in Fig. 1(b)). During the hydrothermal oxidization, the Ti atoms and the inserted Ni ions are *in-situ* oxidized to anatase TiO_2_ and nickel oxide respectively. The as-produced hybrids still keep a laminar structure. 2D carbon sheets with embedded TiO_2_ and NiO nano-particles can be observed from the lateral dimension (Fig. 1(c)). EDS spectrum (Fig. 1(f)) demonstrates that the sheets of composite consisted of Ti, C, O, and Ni elements.

TEM was also carried out for comprehensive structural characterizations. Figure 1(g) shows a cross section image of a particle of the NiO&TiO_2_@C composites, exhibiting the typical structure and morphology of the hybrid. Plenty of oxide nano-particles are observed on the surface and inside each layers. Figure 1(h) shows a few-layer composite of NiO&TiO_2_@C which most probably consists of several carbon sheets coated with plenty of oxide nano-particles. In Fig. 1(i), a monolayer carbon sheet coated with few nano-particles is observed, inset shows SAED pattern confirming the structure of amorphic carbon.

**Figure 2:**
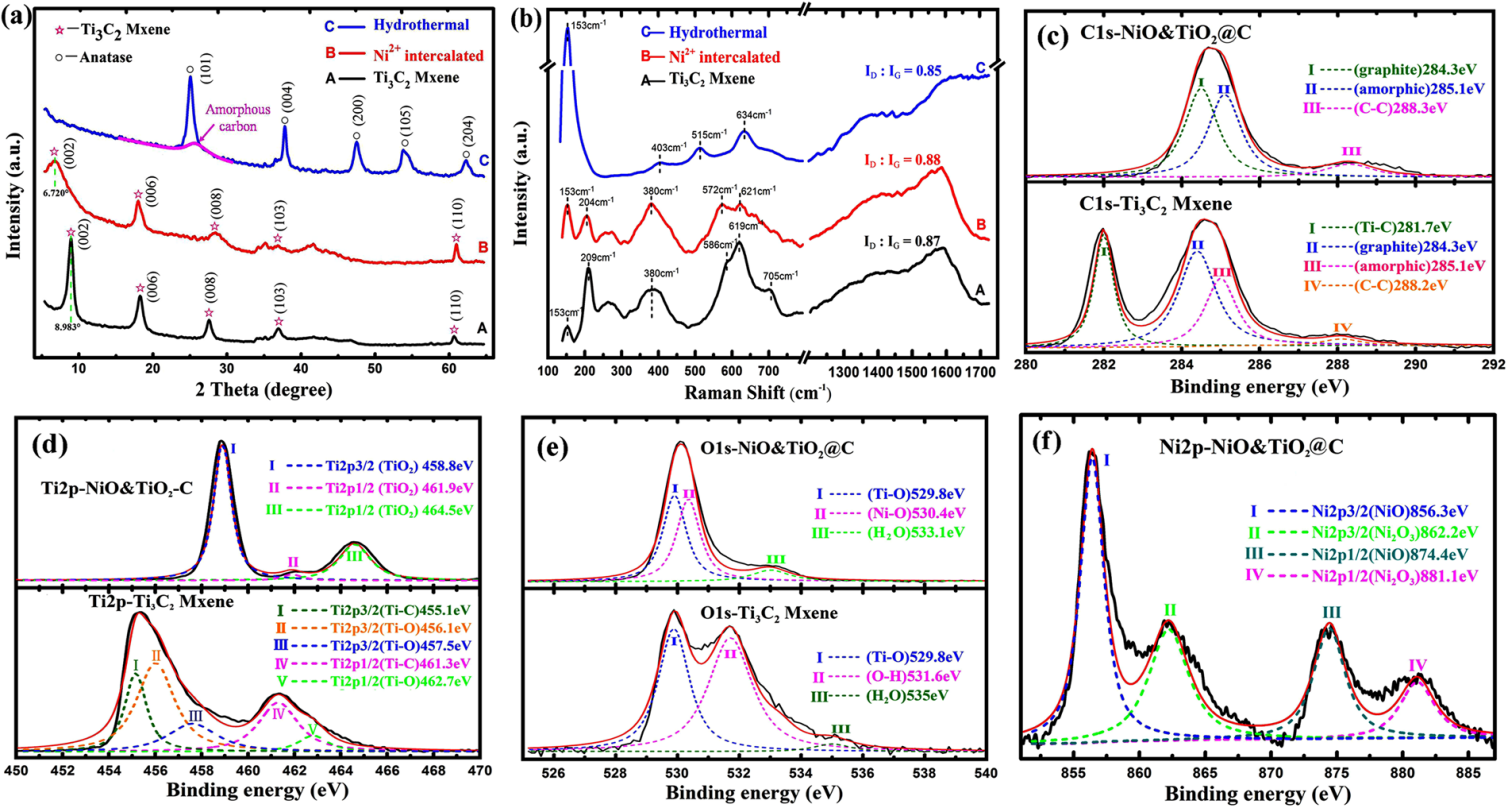
Spectrum of Mxene and Derived Composites: (**a**) XRD patterns and (**b**) Raman spectrum of Ti3C2, Ni^2+^ inserted Ti_3_C_2_ and the as-synthesized hybrid; XPS spectra for (**c**) C1s, (**d**) Ti2p and O1s of pristine Ti_3_C_2_ and the derived NiO&TiO_2_@C composite; XPS spectra for (**f**) Ni2p of NiO&TiO_2_@C composite. (All spectrums are prepared with Origin (OriginLab, Northampton, MA)).

Powder X-ray diffraction (XRD) was conducted to investigate the structure evolution of the as-synthesized composites (Fig. 2(a)). For the original Ti_3_C_2_, the typical diffraction peak (002) appears at around 2θ = 8.98°, indicating that the interlayer distance of Ti_3_C_2_ is about 0.98 nm according to Bragg equation: 2dsinθ = nλ. After Ni^2+^ intercalation, this peak shifted to a lower angle around 2θ = 6.72° as shown in Fig. 2(a), indicating that the c-lattice parameter expanded to about 1.32 nm. In previous studies, it was already well studied that a variety of cations and small molecules, including Li^+^, Na^+^, K^+^, Mg^2+^, Al^3+^ and DMSO, DMF, Amine, etc^13,18,29,30^, can be inserted into Mxene layers. The ion radius of Ni^2+^ is smaller than that of Na^+^ and K^+^, therefore, nickel ions could obviously intercalate into the interlayer of Ti_3_C_2_, if the metal ions and Mxene particles do not chemically react. The driving force which occurs the insertion is that the terminations (-OH and -F) of MXene possess a stronger hydration effect with Nickle ions.

For further details of the as-prepared composites, Raman spectrum and X-ray photoelectron spectroscopy (XPS) were recorded before and after hydrothermal treatment. As can be seen from Fig. 2(b), the Raman spectrum (C curve) of the NiO&TiO_2_@C composite shows a typical anatase structure. This is because the Raman activity of anatase is higher than that of nickel oxide, and the major components of the as-prepared composites is anatase TiO_2_. Raman spectrum of Ti_3_C_2_ (A curve) is well consistent with the previously reported results^13^. The shift at 153 cm^−1^ is believed to be an E_g_ vibration mode of Ti-O bond compared to the results of C-curve^31^. Nickel ion intercalation made the E_g_ vibration of Ti-O bond at 153 cm^−1^ intensified (curve B), for the reason that the expansion in c-direction caused by intercalation could weaken the interaction between the layers, and trigger the delamination which will expose more Ti-O bonds to the surface. The D and G peaks of carbon appears at around 1420 and 1580 cm^−1^. Hydrothermal treatment makes these two peaks broadened, indicating that the carbon sheets of the hybrid are highly disordered amorphous carbon^32^.

A comparison of XPS spectrum of Ti_3_C_2_ and the as-prepared NiO&TiO_2_@C composite provides details concerning chemical composition and surface chemistry. In Fig. 2(c), C1s XPS indicates that the distinct peak of Ti-C bond of Ti_3_C_2_ at 281.7 eV disappeared completely after the hydrothermal treatment, and the peak of amorphic C-C bond intensified. This is well consistent with the previous results of Raman spectrum, that the as-produced carbon sheets are highly disordered under the relatively low temperature of hydrothermal. Ti2p and O1s XPS (Fig. 2(d,e)) results confirm that the peak of Ti-C bond vanished after hydrothermal oxidization, and the peak of Ti-O bond of TiO_2_ intensified, indicating the formation of TiO_2_. Ni2p XPS (Fig. 2(f)) of the composite shows the 2p_3/2_ and 2p_1/2_ doublets at 856.3 and 874.4 eV of NiO, and 862.2 and 881.1 eV of Ni_2_O_3_ respectively, revealing the formation of nickel oxide. A Ni atom ratio of 3.5at% was also obtained from the XPS spectrum, which confirms the previous point that the amount of as-formed nickel oxide is small, and it is undetected by XRD.

**Figure 3:**
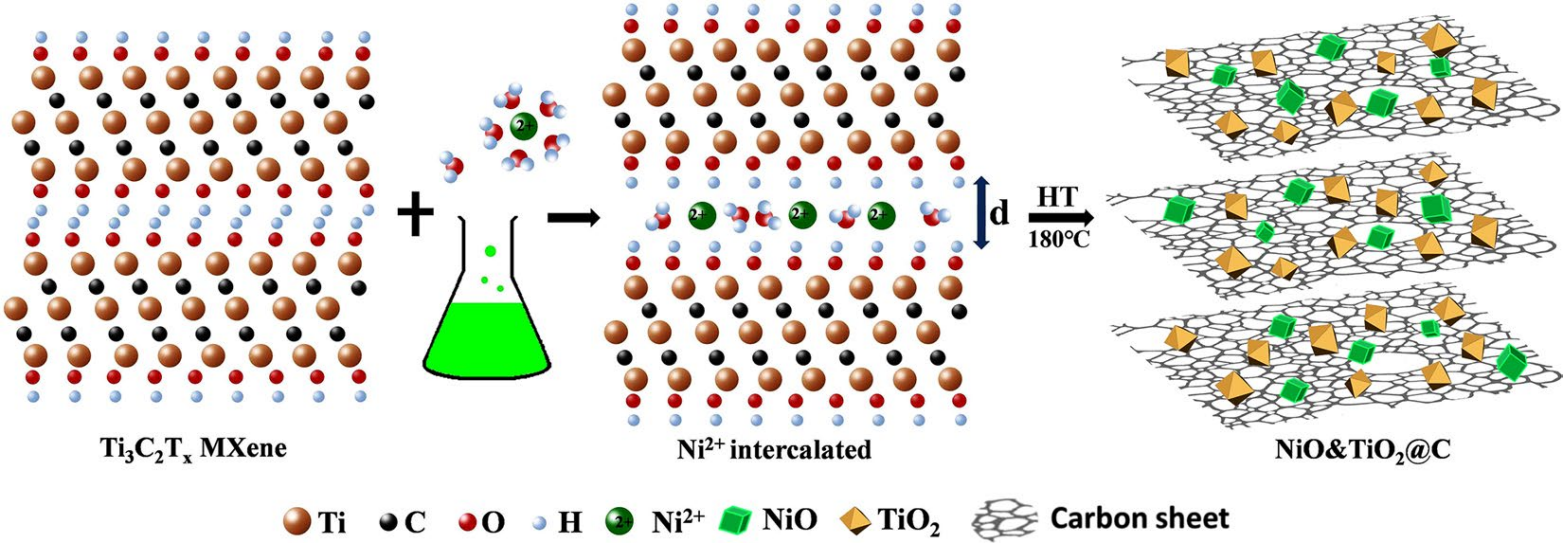
Schematic of Synthetic Process: Schematic of the synthetic process from Ti_3_C_2_ Mxene to NiO&TiO_2_@C composite. (This figure is prepared by Wanlin Feng with Photoshop).

Based on the above results, the structural evolution from Ti_3_C_2_ to NiO&TiO_2_@C can be described as following (showing in Fig. 3). When Ti_3_C_2_ powders are immersed in the green NiCl_2_ solution (1 M), the nickel ions and the water molecules will spontaneously intercalate into the interlayers of Ti_3_C_2_, which results in an expansion in the c-lattice parameter. The following hydrothermal treatment *in-situ* oxidized the intercalated nickel ions and the Ti atoms of Mxene to NiO and TiO_2_ respectively, and finally the amorphous carbon based sandwich-like materials, which is denoted as NiO&TiO_2_@C are formed. The low temperature (180 °C) of hydrothermal makes the as-formed carbon layers highly disordered, and the synthesized oxides could prevent the carbon layers from stacking.

### Electromagnetic responses and Microwave absorbing properties

**Figure 4:**
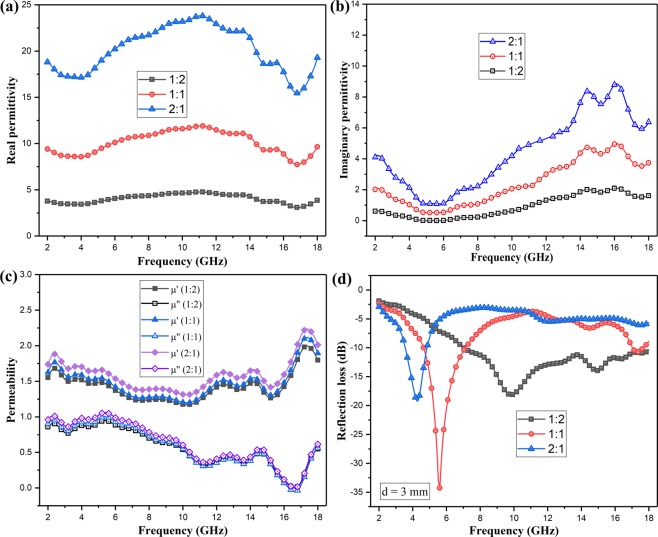
Electromagnetic Parameters: (**a,b**) the real and imaginary parts of the complex permittivity for the composite/paraffin with different mass ratio; (**c**) the complex permeability of the composite/paraffin with different mass ratio; (**d**) Reflection loss of the composite/paraffin with different mass ration at the coating thickness of 3 mm. (All curves are prepared with Origin (OriginLab, Northampton, MA)).

Figure 4(a–c) shows the frequency dependencies of complex permittivity and permeability in the range of 2–18 GHz. Both the real and imaginary part of permittivity of NiO&TiO_2_@C composites climb with the increasing of mass ratio. It is worth noting that the permittivity of the composite/paraffin with the mass ratio of 1:2 are all below 5 with the tested frequency, which are significantly smaller than those of pure Mxene^22,23^. Besides, the real permeability of NiO&TiO_2_@C composites seems to keep around 1.5 within the whole examined frequency range, while the imaginary part decrease gradually from 0.9 to 0.02. It is reasonable that this obvious enhancement of complex permeability of NiO&TiO_2_@C composites compared with that of pure MXene (i.e.μ_r_ ≈ 1) is originated from *in-situ* decoration of NiO. What is more, this synergistic effect of decreased permittivity and enhanced permeability brings them closer, which is quite essential to improve impedance matching. Moreover, both complex permeability and permittivity fluctuate obviously within examined frequency range, which is favorable to microwave attenuation. The derived carbon layers keep the composites a relatively high conductivity. This can be seen from the relatively low real and imaginary part of permittivity. And this could be beneficial to the transmission of microwave energy to heat energy for the enhancement of induced micro current, including current caused by the change of electric field and the eddy current caused by changes of magnetic field.

**Figure 5:**
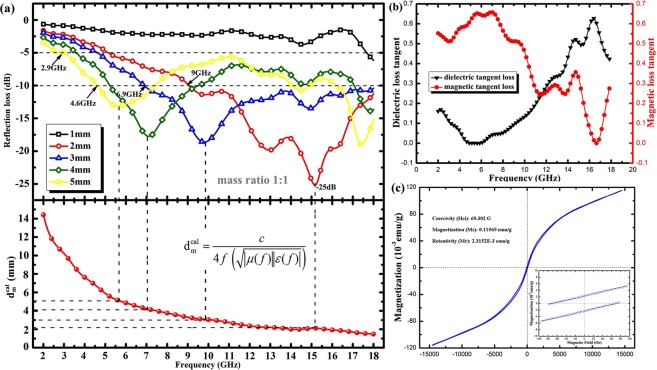
Microwave Absorbing Performance: (**a**) The calculated reflection losses for NiO&TiO_2_@C paraffin wax composite (mass ratio fixed at 1:2) samples with different thicknesses in the frequency range of 2–18 GHz, and the predicted matching thickness d_m_^cal^ at the matching frequency *f*; (**b**) the corresponding dielectric and magnetic loss tangent based on the EM parameters; (**c**) Magnetic hysteresis loop of NiO&TiO_2_@C composites at room temperature (inset is the expanded low field hysteresis curve). (All curves are prepared with Origin (OriginLab, Northampton, MA)).

Figure 4(d) displays the reflection loss of the composite/paraffin with different mass ratio at the fixed coating thickness of 3 mm. Composite/paraffin hybrids with a mass ratio of 1:2 possess the best microwave absorption properties with an effective absorption bandwidth of 11.1 GHz (6.9–18 GHz). Figure 5(a) shows the calculated reflection loss (RL) of composites with NiO&TiO_2_@C over 2~18 GHz at five typical values of thickness with the mass ratio of 1:2. As clearly seen, the RL keeps lower than −10 dB over a broad range of 9 GHz (9–18 GHz) when the thickness of absorber fixes at only 2 mm, which suggests more than 90% of energy of incident EMW has been absorbed. When the thickness is 3 mm, an even broader range of 11.1 GHz (6.9–18 GHz) was achieved, and an optimal RL of −25 dB (corresponding to 99.5% absorption) was also achieved at 15.2 GHz. The RL value is lower than −10 dB within 5.2–9.5 GHz range at 4 mm, and 4.6–7.4 GHz range at 5 mm respectively. Compared to that of pure MXene^23^ and oxidized MXene^24,25^, NiO&TiO_2_@C composites possess broadened effective absorption bandwidth, which confirms that oxidization and magnetism are helpful to attenuate microwave energy here.

Moreover, we noticed that the optimal absorption peak downshifts to lower frequency range when the coating layer gets thicker. According to the resonant absorb theory, when microwave is incident on an absorber sample backed by a perfect conductor, the predicted matching thickness d_m_^cal^ at the matching frequency *f* is given by^33^:

$${{\rm{d}}}_{m}^{cal}=\frac{c}{4f(\sqrt{|\mu (f)\varepsilon (f)|})}$$

where μ(*f*) and ε(*f*) are complex permeability and permittivity at the frequency of *f*, respectively. The calculated d_m_^cal^ based on Eq. (3-1) is shown in Fig. 5(a), which is well consistent with the d_m_^mat^ obtained from reflection loss results. Thus, the attenuation peaks of NiO&TiO_2_@C composites shift to lower frequency with increasing sample thickness. And this result indicates that quarter-wavelength absorption is one of effective way to improve microwave absorption for the modified carbon based composite.

The dielectric loss tangent (tan δ_E_ = ε″/ε′) and the magnetic loss tangent (tan δ_M_ = μ″/μ′) were also calculated based on the permittivity and permeability as shown in Fig. 5(b). The maximum values of both the dielectric and magnetic loss tangent exceed 0.6, suggesting a strong attenuation capability. The collaboration of dielectric and magnetic loss is supposed to be responsible for broadening the microwave absorbing band.

The magnetic responses of NiO&TiO_2_@C composites are believed to be helpful to enhance the microwave absorbing performance. For further confirmation of the magnetic properties of the NiO&TiO_2_@C composite, magnetization curve was examined at room temperature. As shown in Fig. 5(c), there exist small hysteresis loop at lower field at room temperature, indicating the existence of a ferromagnetic component. The saturation magnetization (M_s_), remnant magnetization (M_r_) and coercivity (H_c_) values are 0.11569 emu/g, 2.3152E-3 emu/g and 69.302 G respectively. Since the TiO_2_ particles are non-magnetic, obviously magnetism of the composites originates from NiO nanoparticles. It should be noted that the magnetism of NiO&TiO_2_@C composites is quite weak so that the measuring errors could seriously affect the tested results both the permeability data and magnetism. To minimize the adverse effect caused by measuring errors, we usually prepare 2 samples and test each for 3 times. Furthermore we could not evaluate the contribution of microwave absorption caused by magnetic loss based on the electromagnetic responses and magnetization curves directly. However it can be determined based on the above results that there indeed exists magnetic responses in NiO&TiO_2_@C composites which could help to attenuate microwave energy.

**Figure 6:**
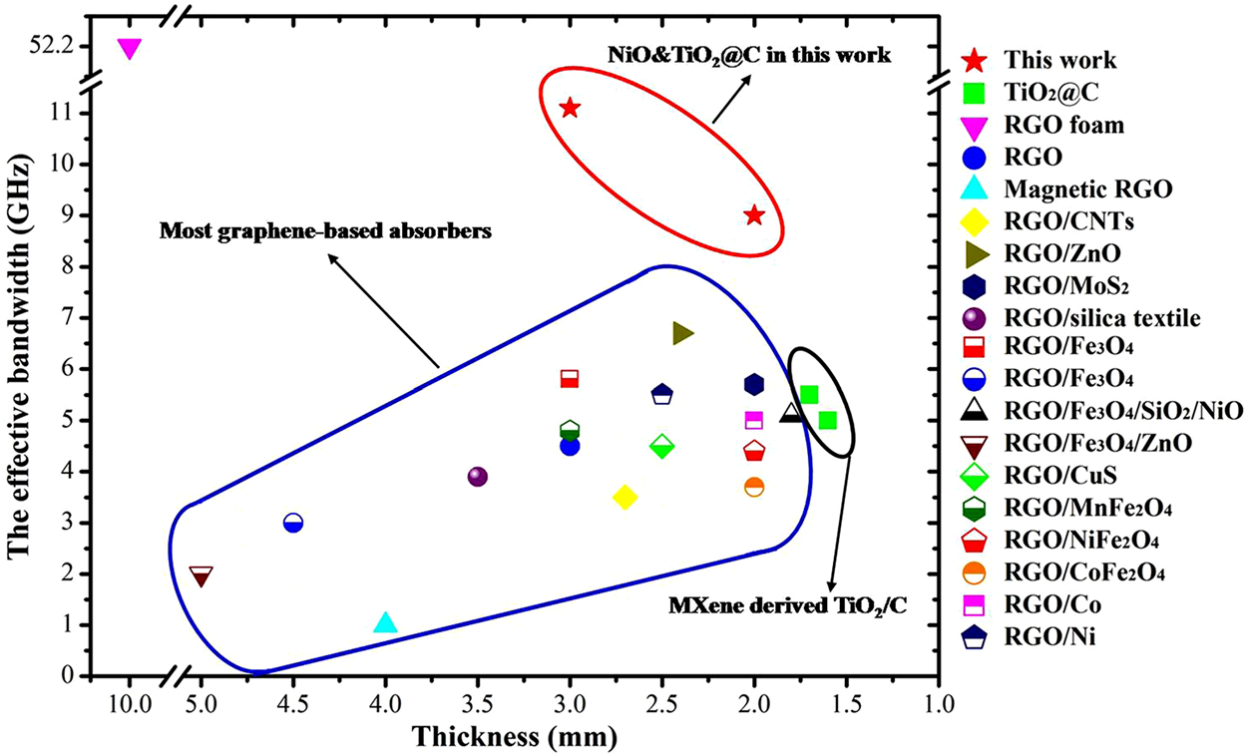
A Comparison with Graphene based Microwave Absorbing Materials: the effective bandwidth versus thickness of typical Graphene-based microwave absorbing materials: TiO_2_/C^25^, RGO^36,37^, RGO foam^3^, RGO/CNTs^12^, RGO/ZnO^38^, RGO/MoS_2_^39^, RGO/SiC^40^, RGO/Fe_x_O_y_^9,41–44^, RGO/CuS^45^, RGO/MFe_2_O_4_^46–48^, RGO/Co^49^, RGO/Ni^50^, and the as-prepared NiO&TiO_2_@C composite in this work. (This picture is prepared with Origin (OriginLab, Northampton, MA)).

Figure 6 exhibits a comparison of EMW absorbing ability between the widely studied graphene-based absorbers and the as-derived NiO&TiO_2_@C composite. As mentioned before, the most commonly used strategy is to assemble magnetic particles with graphene, which were expected to be an effective way to broaden the bandwidth. However, the MA ability of the assembled graphene-based composites are limited by the magnetic properties of the particles and the instinct characteristic of graphene. As can be seen from Fig. 6, the MA ability of most graphene-based composites are in the lower right zone, which means a poor effective bandwidth. Graphene foams show extremely broad bandwidth (>52 GHz)^3^, but it can not be easily applied as a coating material, because of its porous 3D structure. In this work, the as-prepared NiO&TiO_2_@C hybrids exhibit an excellent MA ability: extremely broaden bandwidth at low thicknesses. The main reasons behind are the highly disordered carbon sheets and the combination of multi-attenuation mechanism. The former possesses lots of defects and dipoles that could produce strong dielectric loss, and the later could attenuation EM energy in different ways. Apparently can be seen, the MA ability of NiO&TiO_2_@C composites in this work is much better than that of Mxene-derived TiO_2_/C composites^25^, which indicates that magnetic components plays an important role for microwave absorptions.

**Figure 7:**
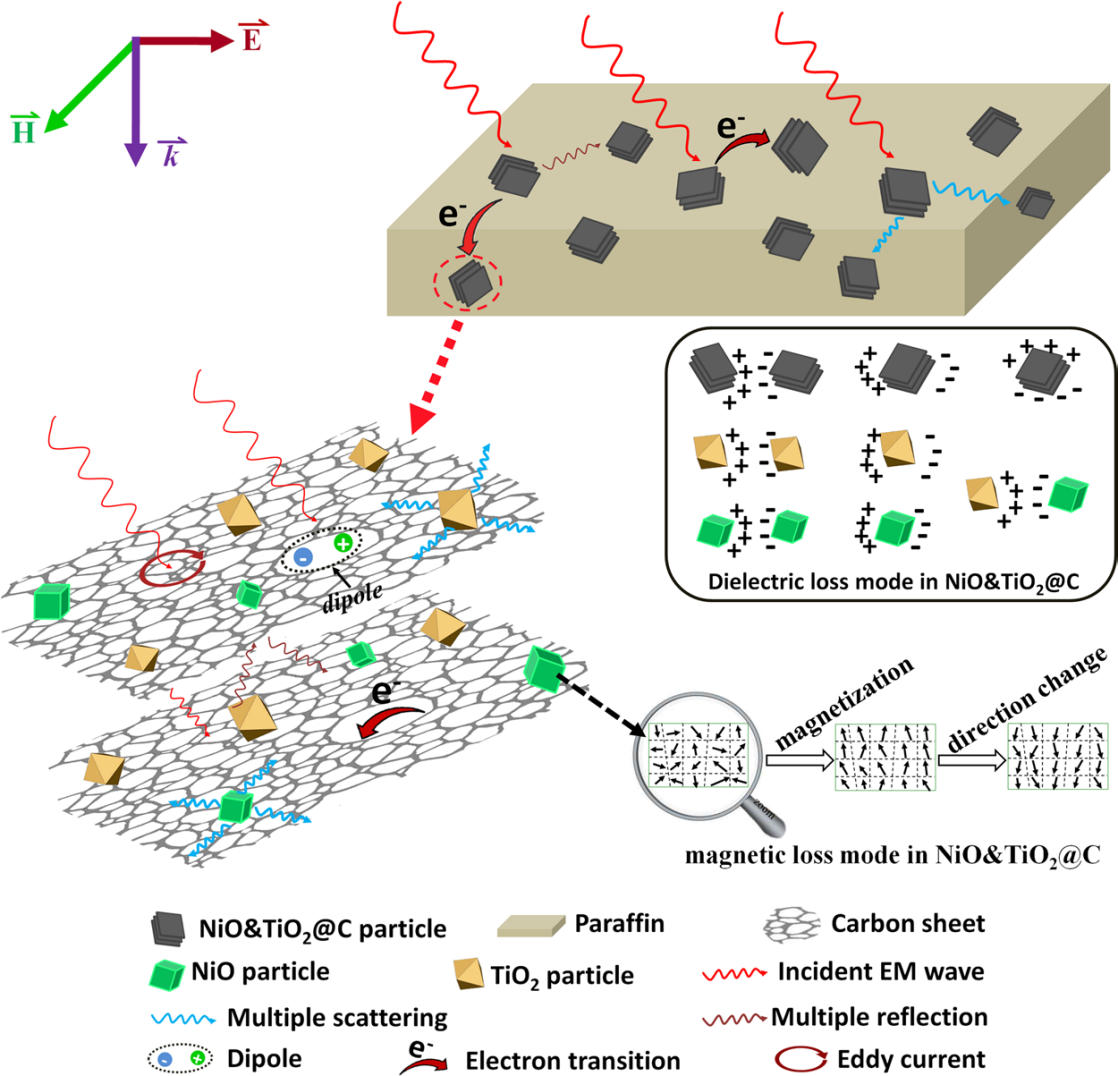
Schematic of Microwave Absorbing Mechanisms: Schematic illustration of microwave absorption mechanisms for NiO&TiO_2_@C composites. (This figure is prepared by Wanlin Feng and Heng Luo with Photoshop).

However, except for the mechanism mentioned above, more attention should be devoted to the mechanisms of intrinsic dielectric loss for the superior microwave absorption performance of the NiO&TiO_2_@C composites. As illustrated in Fig. 7, the superior MA properties could be classified into several aspects. Firstly, the impedance improvement is believed to be beneficial for NiO&TiO_2_@C composites. Due to the relatively higher resistivity and dielectric constant, the *in-situ* produced oxide (TiO_2_ and NiO) nanoparticles could effectively reduce the electrical conductivity of pristine Ti_3_C_2_ (from 1500 S/cm to 15 S/cm)^16,26^, As a result, the composites have a relatively low apparent dielectric constant, which provides a good impedance matching with EMW in air.

Secondly, eddy current effect within highly conductive carbon layers, which induced by alternating incident EMW would allow the conversion of electromagnetic energy into thermal energy. Simultaneously, during the migration procedure of electrons along carbon layer, scattering effect from lattice vibration and inevitable defects^34,35^ on the amorphous carbon layers of as-prepared NiO&TiO_2_@C would also make a contribution to the overall microwave attenuation, leading to electric accumulation around these defects, and as a result, hopping migration. Thirdly, as demonstrated in Fig. 5(b), magnetic loss also makes a contribution to the overall MA performance of NiO&TiO_2_@C composites. Due to the resistance from the inhomogeneity and defects in material, the motion of oriented magnetic domain wall in NiO is inclined to lag behind the incident magnetic field, which in accordance with results in Fig. 5(b). It is this magnetization process that responsible for the conversion of electromagnetic energy into thermal energy. In addition, multiple reflections between the carbon layers, and scattering from the oxide particles could also further enhance the absorbing ability of the composite. In a word, the excellent microwave absorption performance of NiO&TiO_2_@C composites is attributed to the compensatory effect of the three components (NiO, TiO_2_ and amorphous carbon layers) of the composites. From the above discussion, it is highly expected that the NiO&TiO_2_@C composite could be a candidate for high performance microwave absorbing materials.

## Conclusions

A novel laminated and magnetic composite, labeled as NiO&TiO_2_@C, is successfully designed and synthesized via a facile ion intercalation and hydrothermal route. FESEM, TEM, XRD, Raman and XPS characterizations reveal the laminated hybrids composed of amorphous carbon sheets with embedded nickel oxide and anatase TiO_2_ nanoparticles. The composites possess a relatively low permittivity and high dielectric loss as well as a good impedance matching for microwave absorbing materials. The reflection loss (RL) indicates this novel composite has a superior MA ability with a broad range of 6.9–18 GHz (3 mm) and 9–18 GHz (2 mm), respectively while RL is lower than −10 dB. Meanwhile, an optimal RL of −25 dB (corresponding to 99.5% absorption) could be achieved. Furthermore, the excellent microwave absorbing performance is attributed to the compensatory properties of each components. The three components (NiO, TiO_2_ and carbon layer) of the composites could combine the advantages of each part, making the best use of the contributions to microwave absorption. These novel composites have a great potential in developing lightweight, high efficiency and wide range microwave absorbing materials.

## Methods

### Materials preparation

Ti_3_C_2_ was synthesized by etching the aluminum atoms from Ti_3_AlC_2_ powders (commercially available, purity ≥99%, Fosman Scientific company, Beijing) immersed in aqueous HF solution (49 Wt%) in a ultrasonic homogenizer (JY96-IIN, Scientz) and sonicated for 4 h. After the treatment, the mixture was separated by centrifuge and the sediments were washed with deionized water until the pH reached approximately 6.0. Subsequently, the clay-like sediments were immersed in 1 M NiCl_2_ solution to derive Ni^2+^ intercalated Mxene, after which the mixture was sealed in a steel kettle and heated up to 180 °C and held at the temperature for 12 h in an air drying oven to form nickel oxide-titania-carbon (NiO&TiO_2_@C) composites. The final products were washed with distilled water and ethanol for sevral times and then dried at 60 °C under vacuum and denoted as NiO&TiO_2_@C.

### Characterization

The phase composition and crystal structure were confirmed by X-ray Diffraction (XRD) with a powder X-ray diffractometer (D/max-2400, Rigaku) using Cu Kα radiation (λ = 1.54056 Å) and a step scan of 0.02° with 1 s per step. The microstructure was characterized by Scanning Electron Microscopy (SEM) on a Zeiss Supra 50VP, Germany, and TEM operated at 200 k on JEM 2100, Japan; Energy-Dispersive Spectroscopy (EDS) was performed with this instrument (Oxford EDS, with INCA software). The Raman spectrum of all samples were measured on a microspectrometer (inVia, Renishaw plc, Gloucestershire, UK) using an Ar ion laser (514.5 nm) and a grating with 1800 lines mm^−1^. X-ray Photoelectron Spectroscopy (XPS) (using a PHI 5000, ULVAC-PHI, Inc., Japan) was also used to analyze the surface components and their chemical states. Magnetic hysteresis loops were characterized using a vibrating samples magnetometer (Lakeshore 7404) at room temperature.

For the characterization of dielectric responses ranging from 2 GHz to 18 GHz, donut-shaped samples with 7.0 mm outer diameter and 3.0 mm inner diameter were prepared by mixing the as-prepared hybrids powders with molten paraffin (weight ratio equals to 1:2), and the electromagnetic parameters (relative permittivity *ε*_r_ and permeability *μ*_r_) of each sample were determined through coaxial line method with a vector network analyzer (Agilent AV3618).

To evaluate the microwave absorption performance of the prepared NiO&TiO_2_@C composites, the reflection loss (RL) values were calculated based on the relative complex permeability and permittivity at a given layer thickness according to the transmission line theory^33^, as followings:

2-1 $$R{\rm{L}}(dB)=20\,\mathrm{log}|\frac{{Z}_{in}-1}{{Z}_{in}+1}|$$

2-2 $${Z}_{in}=\sqrt{\frac{{\mu }_{r}}{{\varepsilon }_{r}}}\,\tanh [j(\frac{2\pi }{c})\sqrt{{\mu }_{r}{\varepsilon }_{r}}fd]$$

where *Z*_*in*_ is the normalized input impendance, *j* is the imaginary unit, *c* is the speed of light, *f* is the microwave frequency, and *d* is the thickness of the coating layer.
